# Identifying depression in the United States veterans using deep learning algorithms, NHANES 2005–2018

**DOI:** 10.1186/s12888-023-05109-9

**Published:** 2023-08-23

**Authors:** Zihan Qu, Yashan Wang, Dingjie Guo, Guangliang He, Chuanying Sui, Yuqing Duan, Xin Zhang, Linwei Lan, Hengyu Meng, Yajing Wang, Xin Liu

**Affiliations:** 1 Department of Epidemiology and Statistics, School of Public Health, Jilin University, Changchun, 130021 China; 2 School of Computer Science, McGill University, Montreal, H3A 0G4 Canada

**Keywords:** Machine learning, Deep learning, Depression, Veterans, NHANES

## Abstract

**Background:**

Depression is a common mental health problem among veterans, with high mortality. Despite the numerous conducted investigations, the prediction and identification of risk factors for depression are still severely limited. This study used a deep learning algorithm to identify depression in veterans and its factors associated with clinical manifestations.

**Methods:**

Our data originated from the National Health and Nutrition Examination Survey (2005–2018). A dataset of 2,546 veterans was identified using deep learning and five traditional machine learning algorithms with 10-fold cross-validation. Model performance was assessed by examining the area under the subject operating characteristic curve (AUC), accuracy, recall, specificity, precision, and F1 score.

**Results:**

Deep learning had the highest AUC (0.891, 95%CI 0.869–0.914) and specificity (0.906) in identifying depression in veterans. Further study on depression among veterans of different ages showed that the AUC values for deep learning were 0.929 (95%CI 0.904–0.955) in the middle-aged group and 0.924(95%CI 0.900-0.948) in the older age group. In addition to general health conditions, sleep difficulties, memory impairment, work incapacity, income, BMI, and chronic diseases, factors such as vitamins E and C, and palmitic acid were also identified as important influencing factors.

**Conclusions:**

Compared with traditional machine learning methods, deep learning algorithms achieved optimal performance, making it conducive for identifying depression and its risk factors among veterans.

**Supplementary Information:**

The online version contains supplementary material available at 10.1186/s12888-023-05109-9.

## Introduction

As a major human epidemic, depression ranks ninth in terms of total disability and death, following conditions such as heart disease, stroke, and AIDS [1]. It stands as one of the leading causes of disability worldwide, increases the overall global burden of disease [2]. Depressive episodes are characterized by progressive and sudden onset, with variable duration [3], frequency, and mode of occurrence. The risk of occurrence increases with each episode. Furthermore, age is an important influencing factor in depression [4, 5]. The onset and recurrence of depression tend to be detrimental to the prognosis as the age of onset increases [6]. Depression is often not widely diagnosed and treated due to stigma in filling out the depression scale, inadequate mental health resources, and the tendency to conceal depressive symptoms, making the disorder difficult to identify and predict.

Among veterans, the prevalence of major depressive symptoms was 31%, which is two to five times [7] higher than that of the general U.S. population. Military personnel who participated in deployment were twice as likely to develop depression as those who were not deployed (OR = 2.8) [8]. A cohort study suggested that veterans with depression had a higher risk of suicide [9]. In addition to suicide and injury-related causes of death [10], depression is associated with an increased risk of death from nearly all major medical causes. The cohort study of Quinn D Kellerman et al. [11] showed a higher risk of mortality in heart disease, diabetes, hypertension, and cerebrovascular disease among veterans with depression [12].

In the medical field, machine learning has been proven to be highly predictive [13]. Traditional machine learning methods have also been well applied in the field of depression recognition [14]. In recent years, with the continuous improvement of the algorithms, deep learning (a sub-domain of machine learning) has shown superior identified capabilities compared to other traditional machine learning models. A recent study using deep learning algorithms to identify the severity of hazardous drinkers and alcohol-related problems have confirmed the optimal outcome of deep learning algorithm [15]. To date, no study has used deep learning algorithms to identify depression in veterans.

Therefore, we mainly focused on the effectiveness of deep learning algorithms in identifying depression in veterans. By using 10-fold cross-validation, we compared the deep learning models (DL) and five traditional machine learning models: eXtreme Gradient Boosting (XGBoost), Decision Tree (DT), Support Vector Machines (SVM), K Nearest Neighbor (KNN), and Random Forest (RF), as well as the area under the subject operating characteristic curve (AUC), accuracy, recall, specificity, precision and F1 score to evaluate the identification effectiveness of the model. Considering the significant impact of age on depression, we further identify important variables for middle-aged and older veterans by this algorithm and ranked the contributions.

## Methods

### Dataset description

We obtained a total of 2,546 veterans as study subjects in the National Health and Nutrition Examination Survey (NHANES) database. The NHANES database is a long-standing and representative survey conducted by the National Center for Health Statistics (NCHS) [16]. A substantial amount of data, including personal health and nutrition information, biometric data, and laboratory test results, was collected by conducting face-to-face interviews, physical examinations, and laboratory tests. A multi-stage sampling method was used to obtain a representative sample of individuals of different age groups, races, genders, and socioeconomic backgrounds in the United States. In addition, a cross-sectional study design was used to obtain data from a representative sample of the population at a given point in time. These surveys were conducted in cycles, each lasting two years. Approval from the Institutional Review Board was not required due to the publicity of NHANES data [17].

We combined raw data from seven cycles of the NHANES database from 2005 to 2018, obtaining a total of 70,193 participants. To mitigate the effects of multicollinearity, the variables that remained consistent throughout the seven cycles were selected. Furthermore, variables indicating the same disease were merged. For instance, in the case of hypertension, the selection criteria included satisfying any one of the three items [18]: [BPQ20] Ever been told to have high blood pressure; [BPXSY] systolic blood pressure ≥ 140 mm Hg and/or [ BPXDI] diastolic blood pressure ≥ 90 mm Hg; [BPQ040a] Ever been told to take a prescription for hypertension. In the end, we got a total of 755 variables.

### Remove missing values

The values “7”, “77”, “777” and “7777” indicated rejection, while “9”, “99”, “999” and “9999” indicated unknown status and were therefore considered as missing values. Since missing values will affect the predictive classification effect of machine learning [19], all variables with over 20% missing data were excluded, and the remaining variables were filled with missing values through plural interpolation.

### Selecting the study population

**Fig. 1:**
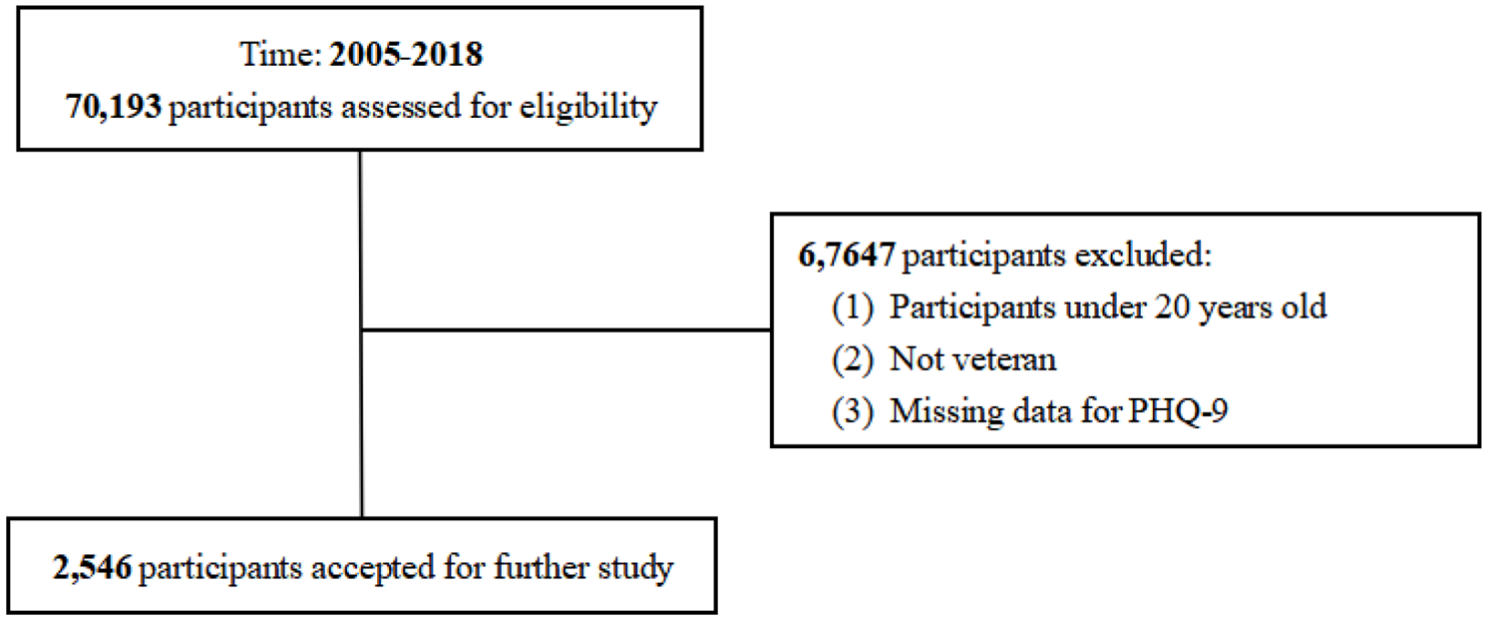
Flowchart of participants selection

Veterans were identified as participants who answered “yes” to the population question (2005–2006 DMQMILIT: Veteran/Military Status; 2007–2010 DMQMILIT: served in the U.S.; DMQMILIT: Served active duty in the U.S. Armed Forces; 2011–2018 DMQMILIZ: Served active duty in the U.S. Armed Forces). Participants who did not answer depression-related questions and those who were under the age of 20 years were excluded from the study. Eventually, a total of 2,546 individuals were included in the study to train the algorithms. (Fig. 1)

### Definition of diseases

The Patient Health Questionnaire-9 (PHQ-9) is the most reliable and validated screening tool for depression in primary health care [20]. It comprises nine questions, with each item scored on a scale range of 0–3, resulting in a total score of 27. Participants with scores ≥ 10 on PHQ-9 were considered to have clinically significant depressive symptoms. Therefore, a threshold of 10 was selected for diagnosing depression [21]. To compare the difference of variables between the depressed group and the non-depressed group, categorical variables were tested by SPSS 24.0 using chi-square tests, and a two-sided *P* < 0.05 was considered statistically significant.

### Model development and validation

The algorithms used in this study were implemented in R4.2.1. The variables were selected based on AIC values through backward stepwise regression in “MASS” package, Eventually, 48 variables were retained for analysis [22] (Supplementary Table 1). All data were divided into a training set and a test set at a ratio of 7:3. Furthermore, the “ROSE” package was used in this study to increase the number of minority category samples by random oversampling to balance the dataset [23]. Each algorithm automatically adjusts its hyperparameter values by utilizing a standardized grid of candidate models from the “cart” package. These hyperparameters were subsequently applied to the training data to optimize the model parameters. Deep learning was performed using the h2o.grid function of the H2O platform. Deep learning of the H2O was based on a multilayer feedforward artificial neural network, which was trained using backpropagation for stochastic gradient descent. The model training involved adjusting various parameters, including the activation function (activation="Tanh”, “TanhWithDropout”, “Rectifier”, “Rectifier with dropout”), the range of hidden layers (hidden = c (20, 20), (40, 40), (100, 100), (30, 30, 30)), input dropout ratio (input_dropout_ratio = c (0, 0.05)), and learning rate (rate = c (0.01, 0.25)). The number of epochs was set to 10 by default to filter the best-performing model.

The other five traditional machine learning algorithms, XGBoost, DT, SVM, KNN, and RF were compared with deep learning in the study. (1) XGBoost is a large-scale machine learning algorithm, first officially released in 2016, that was built iteratively to minimize function loss [24]. (2) DT represents a tree-like structure, where each node corresponds to an attribute, the branches represent decision rules, and the leaf nodes represent output classes [25]. (3) SVM uses a one-two hyperplane to split the data into four kernel functions: linear kernel, polynomial kernel, radial basis function, and sigmoid kernel [26]. (4) KNN algorithm is a simple non-parametric method that customizes the information of its neighboring points and classifies the output labels based on a similarity measure [27]. (5) RF is an integrated classification algorithm consisting of a large number of individual decision trees, which employs bootstrap aggregation and randomization of predictor variables to achieve a high degree of predictive accuracy [28].

To reduce the risk of overfitting and bias, we select the best model and hyperparameter combination by 10-fold cross-validation (Supplementary Table 2). The evaluation was performed based on six metrics: AUC, accuracy, recall, specificity, precision, and F1-score [29]. AUC serves as an evaluation metric that provides a comprehensive measure of model classification performance in both balanced and unbalanced datasets. It remains independent of data distribution, insensitive to classification thresholds, and combines two important metrics: the true positive rate and the false positive rate. Consequently, we utilized the magnitude of the AUC (0.8–0.9 is considered good and above 0.9 is considered excellent [17]) as the primary assessment metric for evaluating model performance. Finally, the importance scores of the variables were obtained, and the contribution ranking was analyzed [30].

## Results

### Classification model performance

Of the 2,546 veterans included in the study from 2005 to 2018, 185 (7.27%) individuals suffered from depression. The demographics and characteristics of the patients are summarized in Table 1. The input variables used to characterize the selected data included gender, age, race, education, marital status, family income to poverty ratio, and BMI (kg/m²). The differences in age, marital status, ratio of family income to poverty and BMI (kg/m^2^) were statistically significant (*P* < 0.05). Among all participants, 2,386 were males (93.7%), and 160 were females (6.3%). The number of young, middle-aged, and elderly individuals were 273(10.7%), 913(35.9%), and 1,360(53.4%), respectively.

**Table 1:** Baseline characteristics of depression in United States veterans

variable		Total (%)	Non-depression	depression	χ^2^	P
Gender	Male	2386(93.7)	2218(93.0)	168(7.0)	2.858	0.091
	Female	160(6.3)	143(89.4)	17(10.6)		
Age	Youth	273(10.7)	243(89.0)	30(11.0)	13.903	**< 0.001**
	Middle age	913(35.9)	834(91.3)	79(8.7)		
	Old age	1360(53.4)	1284(94.4)	76(5.6)		
Race	Mexican American	137(5.4)	124(90.5)	13(9.5)	6.805	0.078
	Non-Hispanic White	1480(58.1)	1388(93.8)	92(6.2)		
	Non-Hispanic Black	666(26.2)	612(91.9)	54(8.1)		
	Other	263(10.3)	237(90.1)	26(9.9)		
Education	<High school	328(12.9)	296(90.2)	32(9.8)	5.098	0.078
	High school	623(24.5)	573(92.0)	50(8.0)		
	>High school	1595(62.6)	1492(93.5)	103(6.5)		
Marital status	Live together	1685(66.2)	1589(94.3)	96(5.7)	18.203	**< 0.001**
	Single	861(33.8)	772(89.7)	89(10.3)		
Ratio of family income to poverty	< 1.3	510(20.0)	438(85.9)	72(14.1)	44.427	**< 0.001**
	≥ 1.3	2036(80.0)	1923(94.4)	133(5.6)		
BMI(Kg/m²)	Under weight	29(1.1)	28(96.6)	1(3.4)	18.969	**< 0.001**
	Normal weight	579(22.7)	538(82.9)	41(7.1)		
	Over weight	968(38.0)	921(95.1)	47(4.9)		
	Obesity	970(38.2)	874(90.1)	96(9.9)		

BMI—body mass index

**Fig. 2:**
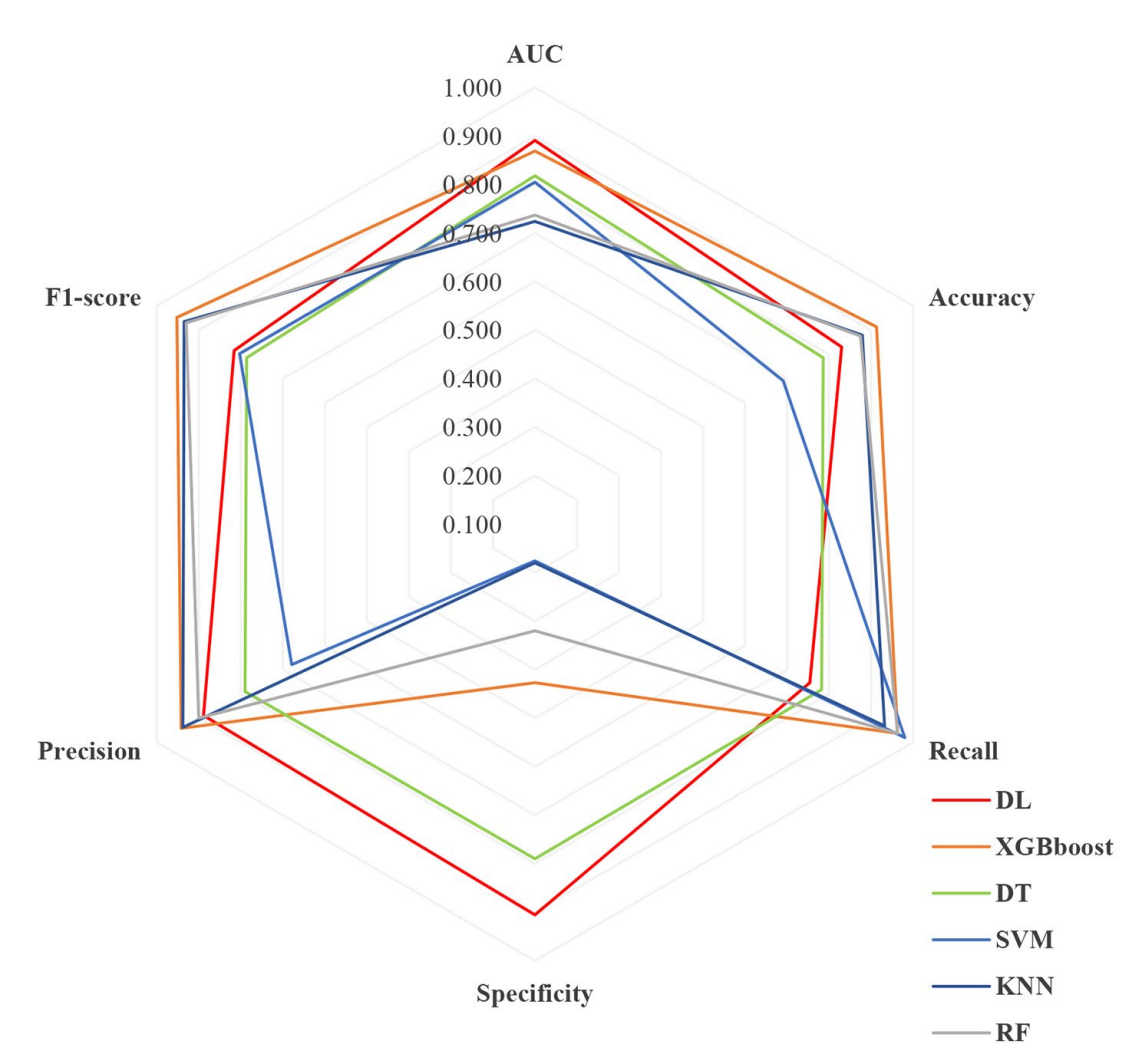
Radar plot of predication abilities for the United States veterans. DL—Deep Learning; XGBoost—eXtreme Gradient Boosting; DT—Decision Tree; SVM—support vector machines; KNN—K Nearest Neighbors; RF—random forests

DL and other traditional machine learning algorithms are used to train the data and select the optimal hyperparameters for a 10-fold cross-validated model evaluation, and the ROC curves are shown in Fig. 2. the six metrics of DL were AUC (0.891, 95%CI 0.869–0.914), accuracy (0.830), recall (0.754), specificity (0.906), precision (0.889), and F1-score (0.816). AUC was selected as the primary evaluation metric. The AUC value of the DL was the highest, while that of other traditional machine models was XGBoost (0.869, 95%CI 0.824–0.915), DT (0.818, 95%CI 0.787–0.848), SVM (0.805, 95%CI 0.748–0.863), KNN (0.724, 95%CI 0.653–0.794), and RF (0.737, 95%CI 0.669–0.804), respectively. In identifying the level of depression for the entire veteran population, DL emerged as the best performing algorithm, followed by XGBoost, while KNN exhibited the lowest performance. There was a significant difference (*P* < 0.05) between DL and other traditional machine learning models, namely XGBoost, DT, SVM, KNN, and RF. However, the classification performance of DL was not significantly better than XGBoost (*P =* 0.389).

**Fig. 3:**
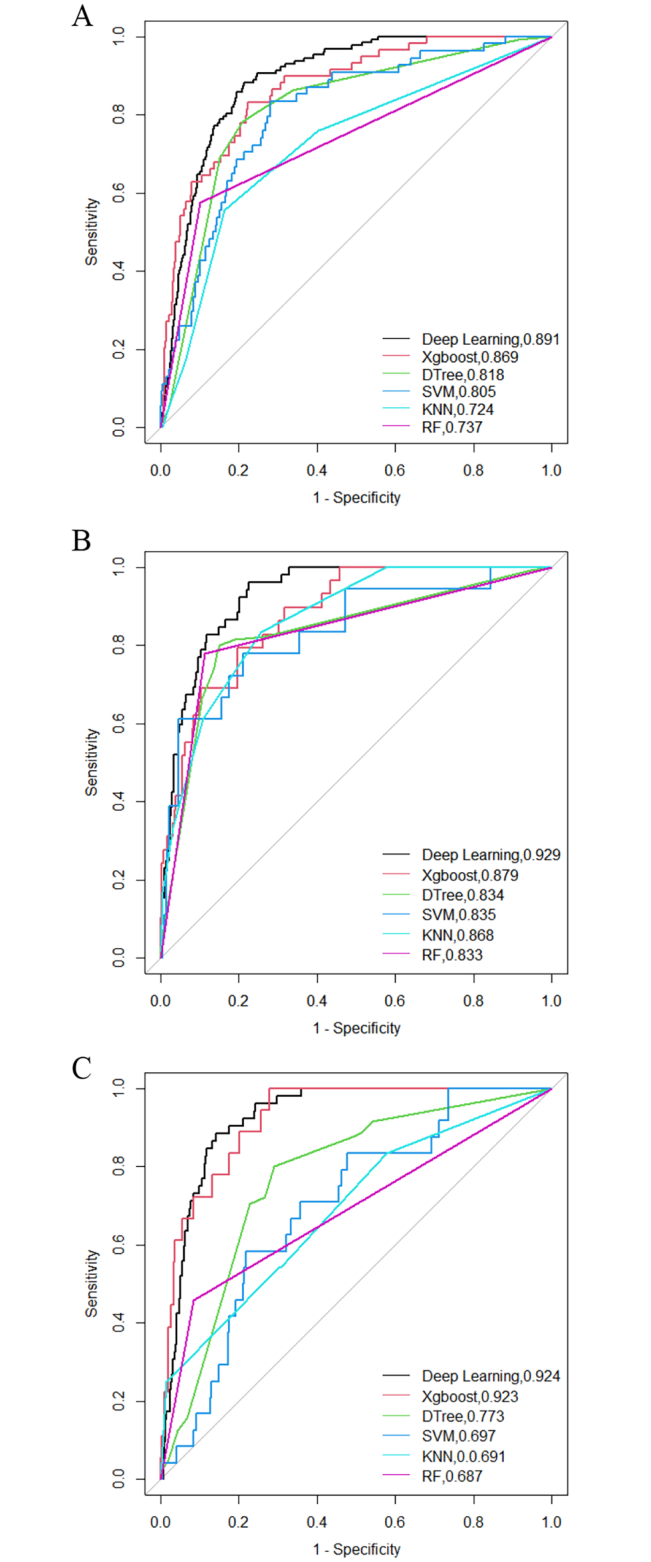
ROC curves for six machine learning models in identifying depression. DL—Deep Learning; XGBoost—eXtreme Gradient Boosting; DT—Decision Tree; SVM—support vector machines; KNN—K Nearest Neighbors; RF—random forests. (**A**) Total Participants. (**B**) Middle-age Participants. (**C**) Older age Participants

In the middle-aged group, DL had the highest AUC (0.929, 95%CI 0.904–0.955), followed by XGBoost (0.879, 95%CI 0.823–0.935) In the elderly group, DL also had the highest AUC (0.924, 95%CI 0.900-0.948), followed by XGBoost (0.923, 95%CI 0.878–0.967). The difference between DL and DT, SVM, KNN, and RF is statistically significant (*P* < 0.05), but not significantly better than XGBoost (*P* = 0.108 for the middle-aged group, *P* = 0.967 for the older age group). The AUC value of DL was stable above 0.900 in different age groups and had the highest specificity and accuracy, which was the best model (Fig. 3; Table 2).

**Table 2:** Six models predict outcomes of depression in middle-aged and older veterans

Index	DL	XGBoost	DT	SVM	KNN	RF
**Total**						
AUC	0.891	0.869	0.818	0.805	0.724	0.737
Accuracy	0.830	0.913	0.786	0.691	0.879	0.875
Recall	0.754	0.963	0.782	0.980	0.932	0.963
Specificity	0.906	0.427	0.790	0.176	0.180	0.320
Precision	0.889	0.942	0.790	0.679	0.938	0.900
F1-score	0.816	0.952	0.786	0.803	0.935	0.930
**Middle age**						
AUC	0.929	0.879	0.834	0.835	0.868	0.833
Accuracy	0.867	0.859	0.816	0.697	0.871	0.880
Recall	0.773	0.965	0.785	0.975	0.966	0.980
Specificity	0.962	0.364	0.852	0.169	0.314	0.359
Precision	0.953	0.877	0.856	0.691	0.892	0.888
F1-score	0.854	0.919	0.819	0.808	0.928	0.932
**Old age**						
AUC	0.924	0.923	0.773	0.697	0.691	0.687
Accuracy	0.860	0.917	0.753	0.702	0.831	0.887
Recall	0.759	0.985	0.802	0.961	0.952	0.961
Specificity	0.960	0.273	0.710	0.122	0.158	0.275
Precision	0.950	0.927	0.711	0.710	0.862	0.917
F1-score	0.844	0.955	0.754	0.817	0.905	0.938

DL—Deep Learning; XGBoost—eXtreme Gradient Boosting;;DT—Decision Tree; SVM—support vector machines; KNN—K Nearest Neighbors; RF—random forests; AUC— receiver operator curve

### Feature importance

The deep learning model was used to calculate the importance scores of the total population of veterans, the middle-aged veterans, and the older veterans (Tables 3 and 4). According to the ranking, the top 20 variables were retained in the total population, and the top three variables were general health conditions (1.000), sleep difficulties (0.963), and memory confusion (0.948). The inability to work due to physical, mental, or emotional problems ranked fourth (0.834). Having an income below 130% of the federal poverty level (i.e., PIR < 1.3) ranked fifth (0.676). In addition to the requirement of special equipment for walking, the diet survey of Vitamin E, palmitic acid, and Vitamin C for the total number of families, BMI, and individuals with some chronic diseases were also important variables affecting the depression of veterans. The number of neutrophils in the biochemical index segment ranked seventh (0.703).

**Table 3:** Identifying the top 20 important variables for overall United States veteran depression through deep learning model

		Code	Label	Importance
Total	1	HUQ010	General health condition	1.0000000
	2	SLQ050	Ever told doctor had trouble sleeping?	0.9631659
	3	PFQ057	Experience confusion/memory problems	0.9480876
	4	PFQ049	Limitations keeping you from working	0.8343042
	5	INDFMPIR	Ratio of family income to poverty	0.7421787
	6	RIDAGEYR	Age in years at screening	0.7248971
	7	LBDNENO	Segmented neutrophils num (1000 cell/uL)	0.7025002
	8	DMDHHSIZ	Total number of people in the Household	0.7007679
	9	MCQ300B	Close relative had asthma?	0.6674766
	10	PFQ054	Need special equipment to walk	0.6487374
	11	HSQ520	SP have flu, pneumonia, ear infection?	0.6469163
	12	MCQ160L	Ever told you had any liver condition	0.6463069
	13	BMXBMI	Body Mass Index (kg/m²)	0.6382723
	14	DR2TATOC	Vitamin E as alpha-tocopherol (mg)	0.6148617
	15	MCQ160D	Ever told you had angina/angina pectoris	0.6122292
	16	Hypertension	Ever told you had high blood pressure or Systolic blood pressure ≥ 140 mmHg, and/or diastolic blood pressure ≥ 90 mmHg.	0.6018993
	17	DR1TS160	SFA 16:0 (Hexadecanoic) (gm)	0.5999041
	18	MCQ160F	Ever told you had a stroke	0.5953889
	19	DR2TVC	Vitamin C (mg)	0.5909083
	20	HSQ510	SP have stomach or intestinal illness?	0.5805472

**Table 4:** Top 15 important variables for middle-aged and older veterans

		Code	Label	Importance
Middle age	1	SLQ050	Ever told doctor had trouble sleeping?	1.0000000
	2	PFQ057	Experience confusion/memory problems	0.8306264
	3	HUQ010	General health condition	0.7768890
	4	HSQ510	SP have stomach or intestinal illness?	0.7510906
	5	MCQ160F	Ever told you had a stroke	0.6759962
	6	KIQ042	Leak urine during physical activities	0.6741676
	7	PFQ054	Need special equipment to walk	0.6403114
	8	PFQ049	Limitations keeping you from working	0.6373989
	9	Hypertension	Ever told you had high blood pressure + Systolic blood pressure ≥ 140 mmHg, and/or diastolic blood pressure ≥ 90 mmHg.	0.6329273
	10	MCQ160A	Doctor ever said you had arthritis	0.6317729
	11	DR1TP226	PFA 22:6 (Docosahexaenoic) (gm)	0.6258965
	12	MCQ160L	Ever told you had any liver condition	0.6164003
	13	DR1TS160	SFA 16:0 (Hexadecanoic) (gm)	0.6159058
	14	MCQ160E	Ever told you had heart attack	0.6116369
	15	HSQ520	SP have flu, pneumonia, ear infection?	0.6103028
Old age	1	HUQ010	General health condition	1.0000000
	2	PFQ054	Need special equipment to walk	0.8548500
	3	PFQ057	Experience confusion/memory problems	0.7185544
	4	MCQ160F	Ever told you had a stroke	0.6913724
	5	SLQ050	Ever told doctor had trouble sleeping?	0.6728851
	6	Hypertension	Ever told you had high blood pressure + Systolic blood pressure ≥ 140 mmHg, and/or diastolic blood pressure ≥ 90 mmHg.	0.6709751
	7	MCQ160K	Ever told you had chronic bronchitis	0.6636364
	8	DR2TATOC	Vitamin E as alpha-tocopherol (mg)	0.6257717
	9	DR2TS120	SFA 12:0 (Dodecanoic) (gm)	0.6235973
	10	KIQ044	Urinated before reaching the toilet	0.6177957
	11	KIQ042	Leak urine during physical activities	0.6157834
	12	HSQ520	SP have flu, pneumonia, ear infection?	0.6091104
	13	HSQ590	Blood ever tested for HIV virus?	0.5971844
	14	MCQ300A	Close relative had heart attack?	0.5970445
	15	MCQ160A	Doctor ever said you had arthritis	0.5952202

The top 15 variables in the middle-aged and older age groups were retained according to the ranking. The top three variables in the middle-aged group were difficulty sleeping (1.000), memory confusion (0.831), and general health condition (0.777). In addition, the intake of docosahexaenoic acid (0.626) was also an important variable. Meanwhile, the top three variables in the older age group were general health conditions (1.000), the requirement of special equipment in walking (0.855), and memory confusion (0.719).

## Discussion

In this study, the AUC of the deep learning model for the overall population and the test set was found to be greater than 0.85 after different age stratification. Deep learning has consistently shown higher performance in identifying depression in veterans compared to traditional machine learning methods.

Deep learning is mainly applied to identify and predict clinical diseases from imaging data. Both image and text-based data can achieve favorable prediction effects. Currently, deep learning algorithms based on textual data (HCET) obtain the best performance in modelling electronic health record data to predict depression compared to traditional machine learning [31]. Here are also studies that predict clinical and genetic biomarkers for antidepressant drugs in major depression by deep learning, among which the MFNN model with three hidden layers (AUC = 0.806) has the optimal prediction performance [32]. These results highlight the efficacy of deep learning in disease prediction, even in scenarios where imaging data is unavailable.

The same is true for our study. Deep learning had the highest AUC (0.891 95%CI 0.869–0.914), accuracy (0.830), recall (0.754), specificity (0.906), precision (0.889), and F1-score (0.816) in identifying the overall veterans. Followed by the XGBoost: AUC (0.869, 95%CI 0.824–0.915), accuracy (0.913), recall (0.963), specificity (0.427), precision (0.942), and F1-score (0.816). DT ranked third (AUC:0.818, 95%CI 0.787–0.848). DL achieved the highest AUC of 0.929 (95%CI 0.904–0.955) and 0.924 (95%CI 0.900-0.948) in the middle-aged and elderly groups, respectively, with the highest specificity (0.962), precision (0.953) in the middle-aged group, with the highest specificity (0.960), precision (0.950) in the older group.

We found that general health conditions, sleep difficulties, and memory confusion were the top three variables affecting depression among U.S. veterans, and the deep learning algorithm ranked them in terms of their contribution to crucial variables. This finding is similar to previous studies, in which Angela M Benavides et al. found that sleep difficulties in veterans were associated with self-reported depression [33]. It is reported that veterans have six syndromes, with syndrome 1 being “cognitive impairment” characterized by attention, memory, and reasoning problems, with symptoms in insomnia, depression, daytime sleepiness and headache [34]. In addition, job restrictions, the ratio of family income to poverty, the total number of families, the need for special equipment to walk, infections, BMI, and some chronic illnesses (asthma, liver conditions, hypertension, stroke, and stomach or intestinal illnesses) are all significant variables influencing the depression of veterans. Notably, we also found that the depression of veterans was associated with the intake of vitamin E and vitamin C, which may be due to the beneficial effects of vitamin E on the oxidation and inflammatory state of individuals, leading to diminished depressive symptoms [35]. Conversely, vitamin C deficiency is associated with adverse emotional and cognitive effects, which may trigger depression [36]. Urinary leakage, arthritis, soft fatty acid, and docosahexaenoic acid intake played a significant role in the middle-aged group. Meanwhile, chronic bronchitis, urinary leakage, HIV infection, and lauric acid intake figured prominently in the elderly group. Among these factors, urinary leakage is also an important factor influencing depression. Some studies have found that urinary leakage was related to certain monoamines, particularly serotonin [37, 38]. A study conducted by Kristen Sueoka et al. based on the Veterans Aging Cohort found that HIV-infected patients were more likely to experience depressive symptoms (OR = 1.38, 95%CI = 1.18, 1.62) [39]. These exemplified the rationale for using deep learning models to identify factors that influence depression in veterans.

The advantage of this study is its novelty as the first study to identify the depression of veterans through deep learning. Compared with other deep learning prediction models, dietary data, and biochemical indicators were incorporated to find as many important factors related to depression in veterans as possible. Some studies have shown that general practitioners can identify 40–50% of actual cases [4]. The discrepancy becomes more evident when considering different age groups, as only 47.3% of late-life depression and 39.7% of mid-life depression were correctly identified. Therefore, the clinical identification of depression in primary care is often suboptimal. Deep learning algorithms may be a supportive tool to identify depression in veterans due to the high morbidity [40, 41], identification difficulty, and increased risk of suicide and [42] death.

This study has several limitations. Firstly, the cross-sectional survey used in our study could only identify significant variables but was unable to verify causality. Secondly, the study was limited to depression among US veterans and the results were based on a balanced dataset. Further research is necessary to validate and extend our findings in a larger and more diverse dataset to better represent the true distribution of depression among veterans. Lastly, while our research findings may contribute to an overall understanding of depression risk among the veteran population, the diversity of individual experiences and length of service is crucial and should be duly considered in individual assessments and care.

## Conclusion

In this study, the deep learning algorithm has good performance in identifying depression in veterans and is a very effective algorithm. Modeling the identification of veterans’ depression through deep learning algorithms can identify veterans’ depression and their risk factors early enough to provide timely intervention and support, optimize resource allocation and ultimately contributing to the improvement of veterans’ mental health.

### Electronic supplementary material

Below is the link to the electronic supplementary material.

Supplementary Material 1: **Supplementary Table 1**. Codebook

Supplementary Material 2: **Supplementary Table 2**. Summary of the parameter values of each model

## Data Availability

Data supporting the findings of this study can be found on the NHANES homepage. (www.cdc.gov/nchs/nhanes.htm)
