# Supplementary material for: Identifying depression in the United States veterans using deep learning algorithms, NHANES 2005–2018
Source: BMC Psychiatry. 2023 Aug 23;23:620. doi: 10.1186/s12888-023-05109-9 (PMC10463693; doi:10.1186/s12888-023-05109-9)
Supplement: Supplementary file 1 — Supplementary Material 1: Supplementary Table 1. Codebook [file 12888_2023_5109_MOESM1_ESM.docx]

# **Supplementary Table 1. Codebook.**

| **Code** | **Label** | **Question/Description** |
| --- | --- | --- |
| RIDAGEYR | Age in years at screening | Best age in years of the sample person at time of hh screening. |
| DMDMARTL | Marital status | Both males and females 14 years -150 years. |
| INDFMPIR | Ratio of family income to poverty | Poverty income ratio (pir) - a ratio of family income to poverty threshold. |
| DMDHHSIZ | Total number of people in the Household | Total number of people in the household. |
| BMXBMI | Body Mass Index (kg/m²) | Body mass index (kg/m²). |
| BPQ080 | Doctor told you - high cholesterol level | {Have you/has sp} ever been told by a doctor or other health professional that {your/his/her} blood cholesterol level was high? |
| Hypertension | BPQ020+Blood pressure measurement | Ever told you had high blood pressure+Systolic blood pressure ≥ 140 mmHg, and/or diastolic blood pressure ≥ 90 mmHg. |
| CDQ001 | SP ever had pain or discomfort in chest | {Have you/has sp} ever had any pain or discomfort in {your/her/his} chest? |
| DRQSDIET | On special diet? | Are you currently on any kind of diet, either to lose weight or for some other health-related reason? |
| PHQ060 | Dietary supplements? | Have you had any of the following since the fasting time in variable phq010. phq010: when was the last time you ate or drank anything other than plain water? [do not include diet soda, black coffee or tea with saccharine or equal.] (note: variable phq010 was not released as part of this data set because of disclosure issues) |
| HSQ510 | SP have stomach or intestinal illness? | Did {you/sp} have a stomach or intestinal illness with vomiting or diarrhea that started during those 30 days? |
| HUQ010 | General health condition | Both males and females 0 years -120 years |
| HSQ520 | SP have flu, pneumonia, ear infection? | Did {you/sp} have flu, pneumonia, or ear infections that started during those 30 days? |
| HSQ590 | Blood ever tested for HIV virus? | Except for tests {you/sp} may have had as part of blood donations, {have you/has he/has she} ever had {your/his/her} blood tested for the aids virus infection? |
| KIQ022 | Ever told you had weak/failing kidneys | {Have you/has sp} ever been told by a doctor or other health professional that {you/s/he} had weak or failing kidneys? do not include kidney stones, bladder infections, or incontinence. |
| KIQ042 | Leak urine during physical activities | During the past 12 months, {have you/has sp} leaked or lost control of even a small amount of urine with an activity like coughing, lifting or exercise? |
| KIQ044 | Urinated before reaching the toilet | During the past 12 months, {have you/has sp} leaked or lost control of even a small amount of urine with an urge or pressure to urinate and {you/he/she} couldn't get to the toilet fast enough?` |
| MCQ010 | Ever been told you have asthma | Capi instruction: if sp age >= 12, display sp name and \s/he\: if sp age < 12, display \you\ and sp name. |
| MCQ160A | Doctor ever said you had arthritis | Has a doctor or other health professional ever told {you/sp} that {you/s/he} . . .had arthritis? |
| MCQ160D | Ever told you had angina/angina pectoris | Has a doctor or other health professional ever told {you/sp} that {you/s/he} . . .had angina, also called angina pectoris? |
| MCQ160E | Ever told you had heart attack | Has a doctor or other health professional ever told {you/sp} that {you/s/he} . . .had a heart attack (also called myocardial infarction)? |
| MCQ160F | Ever told you had a stroke | Has a doctor or other health professional ever told {you/sp} that {you/s/he} . . .had a stroke? |
| MCQ160K | Ever told you had chronic bronchitis | Has a doctor or other health professional ever told {you/sp} that {you/s/he} . . .had chronic bronchitis? |
| MCQ160L | Ever told you had any liver condition | Has a doctor or other health professional ever told {you/sp} that {you/s/he} . . .had any kind of liver condition? |
| MCQ300A | Close relative had heart attack? | Including living and deceased, were any of {sp's/your} close biological that is, blood relatives including father, mother, sisters or brothers, ever told by a health professional that they had a heart attack or angina (an-gi-na) before the age of 50? |
| MCQ300B | Close relative had asthma? | Including living and deceased, were any of {sp's/your} close biological that is, blood relatives including father, mother, sisters or brothers, ever told by a health professional that they had asthma (az-ma)? |
| PFQ049 | Limitations keeping you from working | The next set of questions is about limitations caused by any long-term physical, mental or emotional problem or illness. please do not include temporary conditions, such as a cold [or pregnancy]. does a physical, mental or emotional problem now keep {you/sp} from working at a job or business? |
| PFQ054 | Need special equipment to walk | Because of a health problem, {do you/does sp} have difficulty walking without using any special equipment? |
| PFQ057 | Experience confusion/memory problems | {Are you/is sp} limited in any way because of difficulty remembering or because {you/s/he} experience{s} periods of confusion? |
| WHQ030 | How do you consider your weight | {Do you/does sp} consider {your/his/her}self now to be . . . |
| SLQ050 | Ever told doctor had trouble sleeping? | {Have you/has sp} ever told a doctor or other health professional that {you have/s/he has} trouble sleeping? |
| URXUCR | Creatinine, urine (mg/dL) | Creatinine, urine (mg/dl) |
| LBXSAL | Albumin (g/dL) | Albumin, refrigerated serum (g/dl) |
| LBDSPHSI | Phosphorus (mmol/L) | Phosphorus (mmol/l) |
| LBDSTBSI | Total bilirubin (umol/L) | Bilirubin, total (umol/l) |
| LBDSTRSI | Triglycerides (mmol/L) | Triglycerides, refrigerated (mmol/l) |
| BMXWAIST | Waist Circumference (cm) | Waist circumference (cm) |
| LBXLYPCT | Lymphocyte percent (%) | Lymphocyte percent (%) |
| LBDNENO | Segmented neutrophils num (1000 cell/uL) | Segmented neutrophils num (1000 cell/ul) |
| LBXRDW | Red cell distribution width (%) | Red cell distribution width (%) |
| DR1TFDFE | Folate, DFE (mcg) | Folate as dietary folate equivalents (mcg) |
| DR1TIRON | Iron (mg) | Iron (mg) |
| DR1TS160 | SFA 16:0 (Hexadecanoic) (gm) | SFA 16:0 (Hexadecanoic) (gm) |
| DR1TP226 | PFA 22:6 (Docosahexaenoic) (gm) | PFA 22:6 (Docosahexaenoic) (gm) |
| DR2TTFAT | Total fat (gm) | Total fat (gm) |
| DR2TATOC | Vitamin E as alpha-tocopherol (mg) | Vitamin E as alpha-tocopherol (mg) |
| DR2TVC | Vitamin C (mg) | Vitamin C (mg) |
| DR2TS120 | SFA 12:0 (Dodecanoic) (gm) | SFA 12:0 (Dodecanoic) (gm) |
