# Supplementary material for: Identifying depression in the United States veterans using deep learning algorithms, NHANES 2005–2018
Source: BMC Psychiatry. 2023 Aug 23;23:620. doi: 10.1186/s12888-023-05109-9 (PMC10463693; doi:10.1186/s12888-023-05109-9)
Supplement: Supplementary file 2 — Supplementary Material 2: Supplementary Table 2. Summary of the parameter values of each model [file 12888_2023_5109_MOESM2_ESM.docx]

**Supplementary Table 2. Summary of the parameter values of each model**

| Model | Parameter | Value |
| --- | --- | --- |
| DL | activation | TanhWithDropout |
|  | hidden | [30, 30, 30] |
|  | Input dropout ratio | 0.00 |
|  | rate | 0.01 |
| XGBoost | Max depth | 2 |
|  | eta | 0.1 |
|  | gamma | 0.5 |
|  | Colsample bytree | 1 |
|  | Min child weight | 1 |
|  | subsample | 0.5 |
| DT | minsplit | 20 |
|  | minbucket | 7 |
|  | cp | 0.01 |
| SVM | kernel | sigmoid |
|  | gamma | 4 |
|  | coef0 | 0.1 |
| KNN | k | 5 |
| RF | mtry | 2 |
